# The role of social exposure in predicting weight bias and weight bias internalisation: an international study

**DOI:** 10.1038/s41366-021-00791-9

**Published:** 2021-03-03

**Authors:** Sarah-Jane F. Stewart, Jane Ogden

**Affiliations:** Department of Psychology, University of Surrey, Guildford, GU2 7XH UK

**Keywords:** Epidemiology, Health policy

## Abstract

**Background:**

Whilst the consequences of weight bias and weight bias internalisation (WBI) have been explored, less is known about the factors contributing to their development. Some research has explored the role of social exposure in weight bias and WBI but has been limited in its definition of exposure and focused solely on western countries. The present study therefore aimed to assess the role of social exposure defined in terms of both population and personal exposure in predicting weight bias and WBI, in an international sample.

**Methods:**

Participants (*N* = 1041) from 33 countries, aged 18–85 years completed online measures of demographics, weight bias, WBI, and population and personal social exposure. Population exposure was defined using national obesity prevalence data from the World Health Organisation to classify countries as low (obesity rates ≤19.9%; *n* = 162), medium (20.0–29.9%; *n* = 672) or high prevalence (≥30%; *n* = 192). Personal exposure was defined in terms of personal contact and health and attractiveness normalisation.

**Results:**

Using regression analysis, greater weight bias was significantly predicted by being younger, male, less educated, and personal exposure in terms of normalisation beliefs that thinner body types are healthier and more attractive, greater daily exposure and overall exposure to thinner friends. The strongest predictors of weight bias (adj *R*^2^ = 13%) were gender *(β* *=* −0.24, *p* < .001), and personal exposure in terms of normalisation beliefs that thinner body types are more attractive (*β* = −0.13, *p* = .001). The strongest predictors of WBI (adj *R*^2^ = 6%) were attractiveness normalisation (*β* = −0.23, *p* < 0.001) and participants’ perceived body shape (*β* = −0.27, *p* < 0.001). Population exposure did not predict either weight bias or WBI.

**Conclusions:**

Personal exposure is more important than population exposure in predicting both weight bias and WBI. Findings hold implications for improving the wellbeing and lived experiences of those living with overweight and obesity.

## Introduction

Weight bias describes the negative attitudes embedded in negative stereotypes towards those with overweight and obesity [1, 2], and weight bias internalisation (WBI) describes the application and endorsement of those negative attitudes to oneself which results in self-devaluation [1, 3]. Much research highlights relationships between weight bias, WBI and various health-related outcomes, such as an increase in depressive symptoms, lower quality of life and maladaptive eating behaviour [1, 2, 4–7]. In addition, the prevalence and pervasiveness of weight bias across a number of domains has been well documented, including interpersonal relationships, the media, the workplace, and educational and healthcare settings [2, 8]. Such bias has been shown to be more prevalent in men, compared with women [9, 10], and in younger adults, compared with older adults [11]. Furthermore, it is estimated that 40% of adults in the US with overweight and obesity show WBI [12] and that although WBI is present across the weight spectrum, it is most likely to be present in those with higher body weights [12–16]. Greater WBI is also more common amongst white women compared to men, and those of other ethnic backgrounds [17]. Despite evidence highlighting the consequences and prevalence of weight bias and WBI, the evidence for the factors contributing towards these problems remains limited [18].

One key causal factor is believed to be social exposure to higher body weights, which may play a role in weight bias and can take two forms: exposure at a population level and exposure at a personal level. In terms of population exposure, only three studies to date have conducted multi-national comparison studies of weight bias [9, 19, 20]. One of these studies found evidence for shared weight bias beliefs in small samples from ten countries (*N* = 610), indicating that greater weight bias was associated with lower education levels, more so than participants’ country of residence [20]. Similarly, research also found consistent levels of weight bias across a large sample from four western countries (*N* = 2866), indicating that greater weight bias was predicted by beliefs of behavioural causes of obesity rather than country of residence [9]. Research is therefore needed to assess the role of national obesity prevalence on weight bias, that draws upon large international samples and uses standardised, validated measures.

Research has also focused on the role of personal exposure. This can take several forms including personal contact with others and normalisation processes, which are underpinned by the role of an individual’s own body weight. With regards to weight bias, the contact hypothesis suggests that it is the impact of increased *positive* contact with those who have overweight and obesity that predicts less weight bias [21, 22]. Specifically, research has consistently shown that increased exposure and close contact with those who have overweight and obesity leads to reduced weight bias [21–23]. This could happen via social contagion processes, whereby individuals become more like those they have close personal relationships with over time. This has largely been studied in relation to physical weight changes, [24, 25], but also in terms of attitudes, beliefs and perceptions surrounding body weight [26, 27]. Research has also indicated a role for sociocultural theory in understanding weight bias, in which greater exposure to thin-ideals and attractiveness norms leads to greater weight bias beliefs via social comparison processes [28].

These processes are also used to understand the development of WBI. Much research indicates that WBI is influenced by our close friends and family, via the use of downward social comparisons of an individual’s own body weight to those around them [25, 29]. From this perspective, research has demonstrated that a perceived disparity between an individual’s ideal body size and their actual body size leads to greater body dissatisfaction and WBI through the internalisation of attractiveness norms [30, 31]. For example, WBI depends on the weight status of those upon which an individual draws their social comparisons, whereby having thinner friends leads to overweight females reporting more WBI [32].

Research has also demonstrated that greater exposure to obesity can not only lead to a shift in attractiveness norms, but also increases tolerance and acceptance of obesity [33]. This reflects the impact of personal exposure via normalisation, whereby increased exposure to larger body sizes leads to a ‘recalibration’ as to what is considered to be healthy and normal. For example, using migration data from women moving from Japan to the USA, research indicated that after only 2 months of living in the USA Japanese women perceived their body size to be smaller, likely due to a recalibration of what normal and healthy body sizes are [34]. In addition, it has been shown that young adults who often socialise with those who have overweight or obesity are less able to identify overweight and obese body weights in others [35].

Therefore, whilst research has documented the consequences of weight bias and WBI, less is known about their cause, particularly the role of social exposure. Social exposure can occur at both a population and personal level and research highlights a number of different mechanisms such as personal contact, the impact of friends and family and the role of media. Some research has explored social exposure at a population level but to date its focus has been limited. Further, whilst some research has demonstrated the impact of each component part of personal exposure, no research has yet explored their comparative impact on weight bias and WBI. Therefore, the present study aimed to investigate the role of demographic variables (age, gender, BMI, education), population exposure (defined through national obesity prevalence rates) and personal exposure (defined in terms of personal contact and normalisation) in predicting weight bias and WBI.

## Methods

### Design

This study utilised a cross-sectional design with an international sample. The predictor variables were population exposure (national obesity prevalence: low, medium and high) and personal exposure (weight normalisation, attractiveness norms and personal contact). The outcome variables were weight bias and WBI.

### Participants

A total of 1041 participants were recruited through snowballing methods and via an advertisement on social media, encouraging individuals to share details of this study with those around the world. The only inclusion criteria were that participants were over the age of 18 years. The research team first utilised their international contacts based in various countries to initiate participant recruitment and encouraged them to share the study with their networks.

## Materials

### Measures

All measures used in this study were translated into the following languages by either native or fluent speakers: English, Danish, Spanish, French, German, Polish, Dutch, Swedish, Chinese, Icelandic, Norwegian, Italian and Arabic.

### Demographics

Participants were asked to provide information of their age, gender, country of residence, how long they have resided in that country (in years), nationality, ethnicity and education level. Participants also self-reported their height and weight to calculate BMI.

### Population exposure

Population exposure was defined in terms of the obesity prevalence rates for each country of residence.

#### National obesity prevalence

Global obesity prevalence data from the World Health Organisation (WHO) [36], was extracted to calculate an estimate of national obesity prevalence. Each participants’ country of residence was allocated a prevalence category number according to the WHO prevalence percentage of obesity categories. These were as follows: 0 = low prevalence (≤19.9%; *n* = 162), 1 = medium prevalence (20.0–29.9%; *n* = 672), and 2=high prevalence (≥30%; *n* = 192).

### Personal exposure

Personal exposure was defined in terms of normalisation and personal contact

#### Normalisation

Normalisation was measured via a standardised methodology used in previous research (eg. 32, 36) that presents participants with a selection of photographs [37] of body shapes and sizes across the weight spectrum, that were each assigned a number from 1 (underweight) −10 (class III obesity). Participants were then asked questions relating to: (i) Health normalisation (1 item: ‘Which of the pictures would you say is most typical of a healthy body weight?’) and (ii) Attractiveness normalisation (1 item: ‘Which of the pictures is closest to the body you would most like to have?’). Participants selecting photographs with higher numbers (larger body sizes) indicates the beliefs that a larger body size is healthier or more attractive.

#### Personal contact

Personal contact was measured using the same photographs and numbering system as above [37], but with the following items: (i) Own body shape exposure (1 item: ‘Which of the pictures is closest to how you look?’); (ii) Daily exposure (I item: ‘Which of the pictures best represents the average person that you see on an average day?’); (iii) Friend exposure (I item: ‘Which of the bodies looks most like your close adult friends?’); iv) Family exposure (1 item: ‘which of the bodies look like adult members of your family?’). Participants selecting photographs with higher numbers (larger body sizes) indicates beliefs that a larger body size is most reflective of your body, most like the average person, your friends and your family.

#### Weight bias

There are a multitude of measures designed to assess weight bias which vary in their focus and psychometric properties [38]. For the present study participants completed an amalgamated and modified version of the standardised weight bias scales informed by previous research [39]. This 23-item scale pools the most valid items from the most commonly used standardised measurement scales of weight bias [19, 40–42]. It has two subscales: *dislike’ of people with obesity* (8 items: ‘I don’t have many friends that have obesity’, ‘People with obesity make me somewhat uncomfortable’, ‘I really don’t like people with obesity much’, ‘Most people without obesity would not want to marry anyone who has obesity’, ‘Most people feel uncomfortable when they associate with people with obesity’, ‘people with obesity should not expect to lead normal lives’ and ‘I have a hard time taking people with obesity too seriously’ and ‘If I were an employer looking to hire, I might avoid hiring a person with obesity’) and *negative stereotypes* (15 items: ‘People with obesity tend to be obese pretty much through their own fault’, ‘People with obesity lack willpower’, ‘People with obesity are undisciplined’, ‘Some people have obesity because they have no willpower’, ‘People with obesity are un-clean’, ‘Workers with obesity cannot be as successful as other workers’, ‘People who have severe obesity are usually untidy’, ‘People with obesity are unattractive’, ‘Most people with obesity resent healthy weight people’, ‘People with obesity are lazy’, ‘People with obesity are gluttonous’, ‘People who have little control over their weight probably have little control over the rest of their lives’, ‘People with obesity are self-indulgent’, ‘Nobody needs to have obesity. If they are, it’s probably because they eat too much or don’t exercise enough’ and ‘People with obesity are insecure’). Participants responded to each statement on a 5-point likert scale from 1(strongly disagree)–5(strongly agree). Participants’ scores were summed to create a total score, a higher score indicates higher weight bias (*α* = 0.93).

#### WBI

WBI was measured through the standardised and validated 12-item weight self-stigma questionnaire (WSSQ) [43]. The WSSQ consists of two distinct subscales: *self-devaluation* (six items: ‘I’ll always go back to being overweight’, ‘I caused my weight problems’, ‘I feel guilty because of my weight problems’, ‘I became overweight because I’m a weak person’, ‘I would never have any problems with weight if I were stronger’ and ‘I don’t have enough self-control to maintain a healthy weight’), and *fear of enacted stigma* (six items: ‘I feel insecure about others’ opinions of me’, ‘people discriminate against me because I’ve had weight problems’, ‘it’s difficult for people who haven’t had weight problems to relate to me’, ‘others will think I lack self-control because of my weight problems’, ‘people think that I am to blame for my weight problems’ and ‘others are ashamed to be around me because of my weight’). Participants responded to each statement on a 5-point likert scale from 1(strongly disagree)–5(strongly agree). Participants’ scores were summed to create a total score, a higher score indicates higher WBI (*α* = 0.88).

### Procedure

This study received institutional ethical approval for conducting research with human subjects. This research was advertised as a study investigating beliefs about body weight. No mention was made to weight bias or social exposure in recruitment materials. This was an online study, and therefore participants were able to complete the study using a smartphone, tablet or computer/laptop. Participants used an anonymous web-link to access the survey host platform ‘Qualtrics’ (Qualtrics, Provo, UT). After providing informed consent, participants completed demographic measures. Participants then completed measures of weight bias, WBI and personal exposure (normalisation and personal contact), and were debriefed. Participants were free to terminate the study at any point by exiting the browser. The questionnaire took participants between 10 and 15 min to complete.

### Data screening

Descriptive statistics showing the distribution of each measurement scale are included in Supplementary Table S1. Assessment of the skewness and kurtosis z-scores indicated variables to not meet assumptions of univariate normality, therefore Kendall’s Tau correlations were run. However, all data met assumptions of multivariate normality after examination of the distribution of residuals [44]. There was no evidence of multicollinearity or homoscedasticity. Therefore all data met assumptions for multiple regression analyses.

### Data analysis

The data were analysed using SPSS Statistics (Version 25). Descriptive statistics were run for all variables. To assess the association between demographic variables (age, gender, BMI and education), population exposure, personal exposure, weight bias and WBI, Kendall’s Tau correlations were run. Gender was measured on three levels: (i) male, (ii) female and (iii) other. Data were received for 15 participants who self-classified to be ‘other’. Data from these participants were excluded from the analyses due to lack of power to include as a separate group. To assess the association between gender, weight bias and WBI, a point-biserial correlation was run. Data indicated multivariate normality and met assumptions, therefore four blocked entry multiple regression analyses were used to assess the role of demographics, population exposure and personal exposure in predicting weight bias and WBI.

## Results

### Demographics

The majority of the sample (*N* = 1026) was female (*N* = 748; 72.9%) and white (*N* = 797; 77.7%). The mean age of participants was 33.90 (SD = 12.36), and the mean BMI of participants was 25.90 (SD = 6.97). Most of the sample were educated to degree level (*N* = 785; 76.5%). A full summary of participant demographics is presented in Table 1. An overview of the countries included in each national obesity prevalence category is presented in Table 2.

**Table 1:** Summary of participant demographics.

	All (*N* = 1026)
Age	*M* = 33.90 *SD* = 12.36 Range = 18 – 85
Gender	Male = 278 (27.1%) Female = 748 (72.9%)
BMI	*M* = 25.90 *SD* = 6.97 Range = 16.44 – 78.19
Ethnicity	White = 797 (77.7%) Black = 25 (2.4%) Asian = 145 (14.1%) Other = 59 (5.8%)
Education	Lower Secondary = 29 (2.8%) Upper Secondary = 167 (16.3%) Vocational Qualification = 45 (4.4%) University Bachelor’s Degree = 382 (37.2%) University Master’s Degree = 248 (24.2%) University Doctoral Degree = 155 (15.1%)

**Table 2:** Sampled countries and national obesity prevalence.

Prevalence category	Country of residence	Obesity prevalence (%)	*N*
High prevalence	New Zealand	30.8	79
	USA	36.2	65
	Saudi Arabia	35.4	46
	Jordan	35.5	1
	United Arab Emirates	31.7	1
		Total	**192**
Medium prevalence	Australia	29.0	259
	UK	27.8	241
	Poland	23.1	59
	Netherlands	20.4	36
	Ireland	25.3	27
	Sweden	20.6	24
	Canada	29.4	8
	France	21.6	3
	Germany	22.3	3
	Argentina	28.3	2
	Israel	26.1	1
	Cyprus	21.8	1
	Czech Republic	26.0	1
	Spain	23.8	1
	Belgium	22.1	1
	Portugal	20.8	1
	Barbados	23.1	1
	Mexico	28.9	1
	Albania	21.7	1
	Ukraine	24.1	1
		Total	**672**
Low prevalence	China	6.2	64
	Singapore	6.1	51
	Italy	19.9	18
	Nigeria	8.9	15
	Denmark	19.7	9
	Switzerland	19.5	2
	India	3.9	2
	Congo	9.6	1
		Total	**162**

National obesity prevalence (%) is taken from Global Health Observatory (GHO) data, WHO (2017).

### The association between demographic variables, population exposure, personal exposure and weight bias

To assess the association between gender and weight bias, a point-biserial correlation was run (*r*_pb_ = −0.22, *p* < 0.001). To assess the association between other demographic variables (age, BMI and education), population exposure, personal exposure and weight bias, Kendall’s–Tau correlations were run. A full correlation matrix is presented in Table 3.

**Table 3:** Kendall’s-Tau correlation matrix between weight bias, WBI demographic, population exposure, and personal exposure variables.

	Weight bias	WBI	Age	BMI	Education	National obesity prevalence	Health normalisation	Attractiveness normalisation	Own body shape exposure	Daily exposure	Friends exposure	Family exposure
Weight bias	1.00											
WBI	0.17***	1.00										
Age	−0.14***	−0.02	1.00									
BMI	−0.11***	0.07**	0.28***	1.00								
Education	−0.11***	−0.04	0.30***	0.07**	1.00							
National obesity prevalence	−0.07***	−0.01	0.24***	0.16***	0.14***	1.00						
Health normalisation	−0.17***	−0.03	0.13***	0.23***	0.01	0.07*	1.00					
Attractiveness normalisation	−0.14***	−0.07**	0.21***	0.40***	0.03	0.08**	0.53***	1.00				
Personal contact												
Own body shape exposure	−0.13***	0.09***	0.25***	0.65***	0.06*	0.12***	0.37***	0.50***	1.00			
Daily exposure	−0.07**	−0.02	0.27***	0.17***	0.12***	0.25***	0.32***	0.30***	0.22***	1.00		
Friends exposure	−0.12***	−0.03	0.29***	0.21***	0.07	0.13***	0.32***	0.36***	0.27***	0.45***	1.00	
Family exposure	−0.07**	0.01	0.06**	0.20***	0.03	0.12***	0.21***	0.25***	0.24***	0.28***	0.27***	1.00

*p < 0.05, **p < 0.01, ***p < 0.001

Weight bias was significantly correlated with all variables. Greater weight bias was associated with being younger, being male, having a lower BMI, having lower education levels, and population exposure in terms of residing in a country with low national obesity prevalence. Further, greater weight bias was also associated with personal exposure in terms of increased daily exposure to thinner people and having thinner close friends and family, and normalisation beliefs that thinner body types are healthier and more attractive, having a thinner own body shape.

### The role of demographic variables, population exposure and personal exposure in predicting weight bias

To assess the role of demographic variables, population exposure, and personal exposure in predicting weight bias, a four blocked-entry multiple regression was run. Weight bias was significantly predicted by age, gender, education level, normalisation, daily exposure and exposure to friends. Greater weight bias was predicted by being younger, male and having lower education levels. In addition, greater weight bias was also predicted by personal exposure in terms of participants having greater daily exposure to larger body sizes and having thinner close friends, and normalisation beliefs that thinner body types are healthier and more attractive. There was no significant role for BMI, population exposure, and personal contact variables of own body shape and exposure to family at the multivariate level. Full results from this multiple regression are presented in Table 4.

**Table 4:** Summary of multiple regression analysis for variables predicting weight bias (N = 1007).

	Model 1			Model 2			Model 3			Model 4		
	*β*	95% CI	*p*	*β*	95% CI	*p*	*β*	95% CI	*p*	*β*	95% CI	*p*
Age	−0.12	[−0.25, −0.07]	<0.001	−0.13	[−0.25, −0.07]	0.001	−0.09	[−0.22, −0.04]	0.005	−0.08	[−0.20, −0.01]	0.026
Gender	−0.20	[−10.04, −5.49]	<0.001	−0.20	[−10.01, −5.44]	<0.001	−0.23	[−10.99, −6.45]	<0.001	−0.24	[−11.46, −6.84]	<0.001
BMI	−0.12	[−0.44, −0.14]	<0.001	−0.12	[−0.44, −0.14]	<0.001	−0.04	[−0.26, 0.07]	0.279	−0.05	[−0.36, 0.09]	0.245
Education	−0.08	[−1.86, −0.29]	0.008	−0.08	[−1.84, −0.26]	0.009	−0.09	[−1.87, −0.32]	0.006	−0.10	[−2.00, −0.45]	0.002
National obesity prevalence				−0.02	[−2.22, 1.37]	0.644	−0.01	[−2.17, 1.36]	0.651	−0.02	[−2.36, 1.25]	0.548
Health normalisation							−0.10	[−2.18, −0.34]	0.007	−0.10	[−2.26, −0.32]	0.009
Attractiveness normalisation							−0.14	[−2.72, −0.76]	0.001	−0.13	[−2.73, −0.66]	0.001
Personal contact												
Own body shape exposure										0.05	[−0.45, 1.23]	0.363
Daily exposure										0.08	[0.03, 1.78]	0.043
Friends exposure										−0.11	[−2.28, −0.48]	0.003
Family exposure										−0.02	[−0.82, 0.37]	0.459
*Adjusted R*^*2*^	0.10			0.09			0.13			0.13		
*F* for change in *R*^2^	27.45***			0.21			19.51***			2.97*		
Model Fit	*F* (4,1003) = 27.45, *p* < 0.001			*F* (5,1002) = 21.98, *p* < 0.001			*F* (7,1000) = 21.85, *p* < 0.001			*F* (11,996) = 15.10, *p* < 0.001		

*p < 0.05*,* ***p < 0.001

In the first model, demographic variables significantly contributed to the regression model and explained 9.5% of the variance in weight bias. Adding population exposure did not significantly improve the model. Adding normalisation beliefs significantly improved the model by explaining an additional 3.4% of the variance. Finally, the addition of personal contact variables significantly improved the model by explaining an additional 1.0% of the variance. Model 1 explained the greatest variance in weight bias, with gender being the strongest predictor of weight bias.

### The association between demographic variables, population exposure, personal exposure and WBI

To assess the association between gender and WBI, a point-biserial correlation was run (*r*_pb_ = 0.07, *p* = 0.028). To assess the association between other demographic variables (age, BMI and education), population exposure, personal exposure and WBI, Kendall’s–Tau correlations were run. The correlation matrix is presented in Table 3.

WBI was significantly correlated with BMI, gender, and personal exposure in terms of participants’ own body shape exposure and attractiveness normalisation. Greater WBI was associated with having a higher BMI, larger body shape, and normalisation beliefs that thinner body types are more attractive.

### The role of demographic variables, population exposure and personal exposure in predicting WBI

To assess the role of demographic variables, population exposure and personal exposure in predicting WBI, a 4 blocked-entry multiple regression was run. WBI was significantly predicted by attractiveness normalisation and participants’ own weight exposure. Greater WBI was predicted by personal exposure in terms of participants having a larger body shape and normalisation beliefs that thinner body types to be more attractive. There was no significant role for age, gender, BMI, education, population exposure, and personal exposure variables of health normalisation, daily exposure, and exposure to friends and family at the multivariate level. Full results from this multiple regression are presented in Table 5.

**Table 5:** Summary of multiple regression analysis for variables predicting WBI (*N* = 1007).

	Model 1			Model 2			Model 3			Model 4		
	*β*	95% CI	*p*	*β*	95% CI	*p*	*β*	95% CI	*p*	*β*	95% CI	*p*
Age	−0.07	[−0.11, −0.00]	0.041	−0.07	[−0.11, −0.00]	0.045	−.05	[−0.10, 0.01]	0.129	−0.06	[−0.11, 0.01]	0.077
Gender	0.07	[0.17, 2.94]	0.028	0.07	[0.17, 2.94]	0.028	.05	[−0.30, 2.48]	0.125	0.02	[−0.88, 1.93]	0.466
BMI	0.16	[0.14, 0.32]	<0.001	0.16	[0.14, 0.32]	<0.001	.23	[0.23, 0.43]	<0.001	0.06	[−0.05, 0.23]	0.189
Education	−0.05	[−0.85, 0.10]	0.125	−0.05	[−0.85, 0.11]	0.128	−.05	[−0.85, 0.10]	0.125	−0.05	[−0.81, 0.14]	0.167
National obesity prevalence				−0.00	[−1.09, 1.09]	0.999	−.00	[−1.10, 1.06]	0.971	−0.00	[−1.11, 1.08]	0.980
Health normalisation							.04	[−0.29, 0.84]	0.341	−0.01	[−0.69, 0.50]	0.753
Attractiveness normalisation							−.17	[−1.85, −0.65]	<0.001	−0.23	[−2.31, −1.05]	<0.001
Personal contact												
*Own body shape exposure*										0.27	[0.80, 1.82]	<0.001
*Daily exposure*										−0.01	[−0.59, 0.48]	0.844
*Friends exposure*										0.00	[−0.55, 0.55]	0.994
*Family exposure*										0.01	[−0.31. 0.42]	0.779
*Adjusted R*^2^	0.03			0.03			0.04			0.06		
*F* for change in *R*^2^	8.36***			0.00			9.23***			6.52***		
Model Fit	*F* (4,1003) = 8.36, *p* < 0.001			*F* (5,1002) = 6.68, *p* < 0.001			*F* (7,1000) = 7.49, *p* < 0.001			*F* (11,996) = 7.24, *p* < 0.001		

***p < 0.001.

In the first model, demographic variables significantly contributed to the regression model and explained 2.8% of the variance in WBI. Adding population exposure to the regression did not significantly improve the model. Adding normalisation beliefs significantly improved the model by explaining an additional 1.8% of the variance. Finally, the addition of personal contact variables significantly improved the model by explaining an additional 2.4% of the variance. WBI was best predicted by participants’ own body shape.

## Discussion

This study aimed to investigate the role of demographic variables (age, gender, BMI and education), population exposure and personal exposure in predicting weight bias and WBI. Greater weight bias was predicted by being younger, being male, having lower education levels, and social exposure variables of believing thinner body types to be healthier and more attractive, participants having greater daily exposure to larger body sizes and thinner close friends. Whilst there was a significant association between greater weight bias and population exposure at the univariate level, this relationship was subsumed by other variables at the multivariate level. Results from this study therefore suggest that population level social exposure is less important than personal social exposure in predicting weight bias. However, demographic variables appear to be most important in predicting weight bias. Our findings that greater weight bias is predicted by being male and having lower education levels supports findings from previous multi-national research [9, 20].

Findings demonstrated that like weight bias, greater WBI was also predicted by believing thinner body types to be more attractive. However, unlike weight bias, findings highlighted that greater WBI was predicted by an individual having a larger body shape. This is likely due to the fundamental conceptual interpersonal vs intrapersonal differences between weight bias [1, 2], and WBI [1, 3], respectively. These findings are supported by the notion that a perceived disparity between your ideal body size, and your actual body size leads to greater body dissatisfaction and greater WBI through the internalisation of the thin-ideal [30, 31]. Therefore, whilst anyone can have WBI, it is typically most prevalent amongst individuals who have overweight or obesity [12, 14–17]. These findings therefore lend support to the growing literature highlighting the potential negative impact of thin-idealistic attractiveness norms upon those with greater body dissatisfaction [45–48].

This research is the first of its kind to explore the comparative predictive role of social exposure at the population and personal level on weight bias. Social exposure at the population level appears to be less important than personal social exposure for weight bias. This finding is aligned with research that suggests we are most influenced by those we form close personal relationships with, such as our friends [21–23, 25, 29], rather than acquaintances and colleagues. Having close contact with those who have overweight and obesity has previously been shown to lead to less weight bias [28], and it has been postulated that having increased and affectively positive contact with people who have overweight or obesity is what underlies this relationship [21, 22].

However, given our finding that greater weight bias is predicted by health normalisation, it is possible that weight normalisation processes could at least partly explain the relationship between greater social exposure to larger body sizes and greater weight bias. Our findings suggest that less weight bias is predicted by considering larger body sizes to be most typical of a healthy weight, compared with smaller body sizes. Our findings therefore support the weight normalisation literature, which demonstrates that greater exposure to obesity can lead to a shift in attractiveness norms and acceptance of obesity [33], in addition to a re-calibration as to what is considered healthy and normal [34, 35]. It would therefore be valuable for future research to further investigate this relationship to explore whether normalisation mediates the relationship between social exposure and weight bias beliefs.

It is important to note that this research is not without limitations. Firstly, although our sample is large and spans across many countries, it still consists of predominantly white women, much like most other research investigating weight bias and WBI [12]. Secondly, our sample is highly educated, which could at least partly account for our finding that education was a significant predictor of weight bias. Thirdly, our sample is restricted to those who are computer literate and have access to the internet, and therefore is not nationally representative. Finally, BMI values were calculated on the basis of participants’ self-reported height and weight, which could have been over- and underestimated, respectively [49, 50]. However, due the multi-national nature of this research, it would not have been possible for the researcher to objectively measure these.

Findings from this study hold important implications for fostering our understanding of how best we can support and improve the wellbeing and quality of life of those living with overweight and obesity, given the literature suggesting the negative health-related outcomes associated with both weight bias and WBI [1, 2, 4–7]. Furthermore, understanding more about the relationship between weight bias and weight normalisation is important in lieu of the growing literature suggesting that perceived weight normalisation can have negative implications for weight status misperception and weight management [51, 52].

In particular these findings suggest important roles for personal social exposure in fostering weight bias and WBI. There is an identified lack of high quality multinational research investigating factors contributing to weight bias and WBI [9]. This research works towards addressing that gap by investigating the comparative role of social exposure at both the population and personal level. However, research in this area is still sparse, and therefore future research should build upon this research using large, diverse populations inclusive of both eastern and western samples. Research in this area is important to provide a deeper understanding of the factors which lead to the development and maintenance of weight stigmatising beliefs both towards the self and to others. Doing so, will help us to better understand how we can best support and improve the wellbeing and quality of life of those living with overweight and obesity.

To conclude, the present study is the first to test demographic variables, population social exposure, and personal social exposure together, to investigate their comparative role in predicting weight bias and WBI. Although weight bias was related to BMI, population exposure, and personal exposure variables of own weight exposure and family exposure at the univariate level, these variables became subsumed at the multivariate level. Therefore, greater weight bias can be best predicted by being younger, male, being less educated, and personal exposure variables of normalisation, greater daily exposure to larger body sizes and having thinner friends. Whilst WBI was related to BMI at the univariate level, this was subsumed at the multivariate level. Greater WBI can best be predicted by personal exposure variables of attractiveness normalisation; participants believing thinner body types to be more attractive, and participants having a larger body shape. These findings better our understanding of the complex factors that contribute to the development and maintenance of weight bias and WBI, which in turn can be used to enhance our understanding of how to improve the lived experiences of those living with overweight and obesity.

## Supplementary information

Supplementary Materials
