# Supplementary material for: The role of social exposure in predicting weight bias and weight bias internalisation: an international study
Source: Int J Obes (Lond). 2021 Mar 3;45(6):1259–70. doi: 10.1038/s41366-021-00791-9 (PMC8159729; doi:10.1038/s41366-021-00791-9)
Supplement: Supplementary file 1 — Supplementary Materials [file 41366_2021_791_MOESM1_ESM.docx]

**Supplementary materials**

Supplementary Table 1.

*Descriptive statistics for outcome measures for all participants, and by national obesity prevalence category.*

|  | Prevalence group | M(SD) | Skewness z-score | Kurtosis z-score |
| --- | --- | --- | --- | --- |
| Weight bias | All | 52.31(17.07) | 5.01 | -1.91 |
|  | Low | 58.36(18.62) |  |  |
|  | Medium | 50.89(16.37) |  |  |
|  | High | 52.35(16.98) |  |  |
|  |  |  |  |  |
| WBI | All | 34.65(10.09) | -4.34 | -1.28 |
|  | Low | 34.49(11.46) |  |  |
|  | Medium | 34.73(9.90) |  |  |
|  | High | 34.70(9.11) |  |  |
|  |  |  |  |  |
| Health normalisation | All | 2.97(1.30) | 28.30 | 48.41 |
|  | Low | 2.80(1.29) |  |  |
|  | Medium | 2.99(1.29) |  |  |
|  | High | 3.03(1.33) |  |  |
|  |  |  |  |  |
| Attractiveness normalisation | All | 2.55(1.36) | 24.70 | 37.90 |
|  | Low | 2.23(1.03) |  |  |
|  | Medium | 2.60(1.44) |  |  |
|  | High | 2.63(1.33) |  |  |
|  |  |  |  |  |
| Personal weight exposure |  |  |  |  |
| Own body shape exposure | All | 3.94(2.09) | 12.31 | 3.65 |
|  | Low | 3.19(1.68) |  |  |
|  | Medium | 4.05(2.15) |  |  |
|  | High | 4.16(2.02) |  |  |
|  |  |  |  |  |
| Daily exposure | All | 4.13(1.43) | 10.41 | 10.46 |
|  | Low | 3.34(1.38) |  |  |
|  | Medium | 4.19(1.38) |  |  |
|  | High | 4.61(1.38) |  |  |
|  |  |  |  |  |
| Friends exposure | All | 3.45(1.42) | 13.86 | 14.90 |
|  | Low | 3.00(1.27) |  |  |
|  | Medium | 3.49(1.40) |  |  |
|  | High | 3.67(1.56) |  |  |
|  |  |  |  |  |
| Family exposure | All | 4.09(1.84) | 10.87 | 3.98 |
|  | Low | 3.36(1.35) |  |  |
|  | Medium | 4.20(1.88) |  |  |
|  | High | 4.35(1.92) |  |  |
